# Medicinal plants sold for treatment of bacterial and parasitic diseases in humans in Maputo city markets, Mozambique

**DOI:** 10.1186/s12906-019-2809-9

**Published:** 2020-01-23

**Authors:** Filomena Barbosa, Delfina Hlashwayo, Victor Sevastyanov, Valeriano Chichava, Adilência Mataveia, Ernesto Boane, Aida Cala

**Affiliations:** 1 Departamento de Ciências Biológicas, Faculdade de Ciências, Universidade Eduardo Mondlane, Avenida Julius Nyerere nr 3453, Campus Principal, Maputo, Mozambique; 2 Departamento de Química, Faculdade de Ciências, Universidade Eduardo Mondlane, Avenida Julius Nyerere nr 3453, Campus Principal, Maputo, Mozambique; 3 Direcção de Ciências Animais, Instituto de Investigação Agrária de Moçambique, Avenida de Moçambique Km 1.5, Maputo, Mozambique

**Keywords:** Medicinal plants, markets, Maputo, Mozambique

## Abstract

**Background:**

In Mozambique, bacterial and parasitic diseases contribute to a high burden of mortality and morbidity. These infectious diseases are treated with antibiotics, antihelmintic or antiparasitic drugs. However, misuse of these has been affecting the potential to treat ailments. It has been reported that many people from Maputo city and province apart from the existing contemporary medicine, still use medicinal plants for treatment of diseases due to traditional heritage and beliefs. It is, therefore, important to register this knowledge in order to use it for future pharmacological studies. This study aimed to identify the medicinal plants sold in Xipamanine, Xiquelene and Mazambane markets for treatment of bacterial and parasitic diseases.

**Methods:**

An ethnobotanical survey, using interviews, was conducted to the main vendors of the markets. Data about the plant name, part used, mode of preparation and administration route were collected.

**Results:**

A total of 64 medicinal plants belonging to 32 families were listed as sold for treatment of bacterial and parasitic diseases in the three markets. *Terminalia sericea*, *Elephantorrhiza elephantina, Tiliacora funifera* and *Hypoxis hemerocallidea* were the most cited plants. Roots were the most often sold suggesting it is the most used part. We also found out that medicinal plants trade is still common in Maputo markets. This suggests that people still use plant-based herbal medicines for their basic health care.

**Conclusions:**

Several medicinal plants were sold in Maputo city’s markets for treatment of bacterial and parasitic diseases, with more emphasis on diarrhea and helminthiases. These plants were commonly bought by local residents and play an important role in the subsistence of vendors. Pharmacological studies are needed in order to isolate the plants active principles and understand their mechanism of action, so that new drugs can be developed.

## Background

Mozambique is a developing country in the Eastern region of Sub-Saharan Africa. According to the World Health Organization (WHO), almost 80% of the population in developing countries depends mainly on traditional medicine for treatment of diseases [1]. This scenario is also observed in Mozambique, where the majority of population (70%) lives in the rural areas [2]. The national healthcare system cannot cover the entire population and some medicines are expensive. Therefore, the population adheres to traditional medicine services [3, 4].

More than 5500 plant species are available in Mozambique and almost 10% are used in the traditional medicine [5]. The trade of medicinal plants in Maputo is known since 1980’s when only 10 traders were found in Xipamanine market [6]. Nowadays, 192 vendors are registered in the Association of Traditional Remedies Vendors from Mozambique (AVEMETRAMO: Associação dos Vendedores de Medicamentos Tradicionais) (unpublished data from 2017). These vendors sell medicinal plants and other non-herbal remedies in the main three medicinal plant markets, namely: Xipamanine, Xiquelene and Mazambane (also known as Adelino) [6].

Bacterial and parasitic diseases have a high burden in Mozambique [7]. The most common bacterial infections cause tuberculosis and diarrhea [7]. Diarrhea, a common symptom of intestinal infection by bacteria and other microorganisms, is among the main causes of morbidity and mortality in children under 5 years old in the country [8]. On the other hand, intestinal parasites, including helminthes and protozoa constitute a major cause of morbidity and mortality in the country [9], where about 11.730.145 children require preventive chemotherapy for soil transmitted helminthiases [10].

Many medicinal plant species have been cited by the vendors of the three markets in a previous ethnobotanical study [6]. With the upcoming antibiotic resistance of many drugs, there is a need for a continuous register of the sold plants, in order to conduct pharmacological studies aiming to develop new and effective drugs. It is likewise important to understand whether medicinal plants are still traded in these markets.

In addition to the advantages described above, there is a need to record existing ethnobotanical knowledge in the markets, to avoid erosion of knowledge. The identification of medicinal plants traded in the markets also gives an indication about the conservation status of species under trade. This type of study also helps to inform the current adherence of the population to the traditional medicine services.

The aim of this study was to identify the medicinal plants sold for treatment of bacterial and parasitic diseases in the three medicinal plant markets in Maputo city, as well as to register data regarding plant names, used parts, mode of preparation and administration routes.

## Methods

### Study site

The study was conducted in the three main medicinal plants trade markets in Maputo city, namely: Xipamanine, Xiquelene and Mazambane.

The city administratively constitutes a Municipality with an elected government and also has the status of Province since 1980. This municipality is divided into seven Municipal districts, namely KaMpfumo, Nlhamakulu, KaMaxakeni, KaMubukwana, KaMavota, KaTembe and KaNyaka. These districts are subdivided into neighborhoods for a total of 63. Territorially, it is the smallest province of the country of 346.77 Km^2^. It currently has 1,101,170.3 inhabitants according to 2017 census, with one of the highest population densities of the country, with 670.6 inhabitants / Km^2^ [11].

The high population density in less urbanized districts combined with poor housing conditions and lack of basic infrastructure make these districts or a part of their neighborhoods the most vulnerable to infectious diseases [12], which are still treated through medicinal plants by many social groups [6].

### Ethical compliance

No ethical approval was obtained because there was no mechanism in place to obtain such approval in the country for studies that do not involve a prospective assessment, laboratory animals and invasive species. Nevertheless, the study complied with the International Society of Ethnobiology (ISE) Code of Ethics [13] and the local legislation on traditional knowledge [14]. The project was also authorized by the Department of Biological Sciences of Eduardo Mondlane University. Approval to conduct research in the markets was obtained from Maputo City Council (*Conselho Municipal de Maputo*) (credential number 105/2018). Prior to data collection, the project was explained to the markets managers in order to tell the objectives of research and to guarantee the safety of indigenous knowledge. The managers gave support in introducing investigators to the “leaders” of the vendors. The aim of the study was clearly explained and all interviewed vendors were asked for their prior oral consent.

### Collection of ethnobotanical information

Ethnobotanical data was collected between January and February 2019 for two weeks. The research team went to Xipamanine market where the data was collected in the first week because it has the majority of medicinal plant vendors. Mazambane and Xiquelene markets were visited in the second week. Snow ball sampling was used. In Xipamanine and Xiquelene markets, the managers referred us to one of the “leaders” (so called because they have more experience in selling medicinal plants), which forwarded us to other “leaders”. This procedure was repeated until we reached the last leader vendor. There are many vendors in the markets, but few have authorization to speak, possibly due to few experience in trade and because they are still learning from the leaders. In Mazambane market, we interviewed the only two vendors of medicinal plants. Using semi-structured interviews, vendor’s socio-demographical information and data related to medicinal use of plants and other remedies were captured. Interviews were made in local language (*Xitsonga/Xichangana*) or Portuguese according to the informant’s preference. In total, 15 interviews were carried out in the three markets. Personal information included: name, gender, age, place of birth, nationality, education levels, how they acquired knowledge about medicinal plants, if they had training on adequate storage of medicinal plants and years of experience on trade. Data was collected regarding plants traded, prices, place of harvest, local names, disease treated, preparation method and administration routes.

### Plant collection and identification

Plant species sold or part of them were registered in local language and identified locally by the botanical technicians and compared with voucher from the University’s herbarium (LMU), Maputo - Mozambique. Unknown plants specimen and/or parts sold were purchased and vouchers were made from those. These vouchers were deposited at the Eduardo Mondlane University’s LMU Herbarium, Maputo - Mozambique. Plant identification was done through vernacular name in *Xitsonga/Xichangana* [15] and through purchased plant materials by botanical technicians from Eduardo Mondlane University, namely: Mr. Ernesto Boane, Mr. Ernesto Nacamo and Eng. Aurélio Bechel. The plant names were confirmed through http://www.theplantlist.org at June 5, 2019. Botanical families followed Angiosperm Phylogeny Group (APG) IV system [16].

### Data analysis

Quantitative analysis of ethnobotanical data was done by calculating relative frequency of citation (RFC) and use value (UV) for all identified plants. Fidelity level (FL) was calculated for the most cited plants.

RFC was calculated by equation [RFC = FC/N] where FC is the frequency of citation of the mentioned species and N is the number of the interviewees [17]. The UV was computed by the number of uses mentioned by each informant for a specie (Ui) divided by the number of interviewees (N) [UV = Ui/N] [18]. Fidelity level was calculated by the number of informants that reported the use of a specie to treat a particular disease (Np) divided by the number of informants that cited the use of the specie for any finality (Ns) [FL = Np/Ns] [19].

## Results

### Sociodemographic information

The majority of vendors were from Xipamanine and Xiquelene markets. Mazambane market had more sellers but many abandoned the place due to the construction work done in the market, which reduced the available space and subsequently led to the payment of a fee for the sale. On the other hand some vendors died. Only one vendor was female. Most of trader had the aged between 50 and 59 years and attended between 6th and 9th grade. They learned about medicinal plants from their families (mother, father, grandparents, sisters and brothers) and only one stated that learned from spirits that taught him through dreams. All vendors were Mozambicans. Almost half (53.3%, *n* = 8) of the vendors attended a training in adequate storage conducted by Direcção Nacional de Medicina Tradicional e Alternativa, the former Instituto de Medicina Tradicional from the Minister of Health. Most of the vendors had between 10 and 34 years of experience in selling medicinal plants. This suggests that they have extensive knowledge in medicinal plants. Detailed socio-demographic data is on Table 1.

**Table 1:** Sociodemographic information of the vendors

	N (%)
Market	
Xipamanine	6 (40.0%)
Xiquelene	7 (46.7%)
Mazambane	2 (13.3%)
Gender	
Male	14 (93.3%)
Female	1 (6.7%)
Age range	
29–39	5 (33.3%)
40–49	4 (26.7%)
50–59	6 (40.0%)
Place of birth	
Gaza	9 (60.0%)
Inhambane	2 (13.3%)
Maputo	4 (26.7%)
Schooling	
No schooling	1 (6.7%)
1-5th grade	4 (26.7%)
6-9th grade	6 (40.0%)
10-12th grade	4 (26.7%)
Learning about medicinal plants	
Family	12 (80.0%)
Self/ spirits	1 (6.7%)
Other vendors	2 (13.3%)
Training on adequate storage	
Yes	8 (53.3%)
No	7 (46.7%)
Years of experience in trading medicinal plants	
10–34	12 (80.0%)
35–50	3 (20.0%)

### Medicinal plants sold in the markets

A total of 64 plants were listed as sold for treatment of bacterial and parasitic diseases in the three markets. Fourteen plants, although mentioned by interviewers, were not identified because they were not available during data collection.

**Fig. 1:**
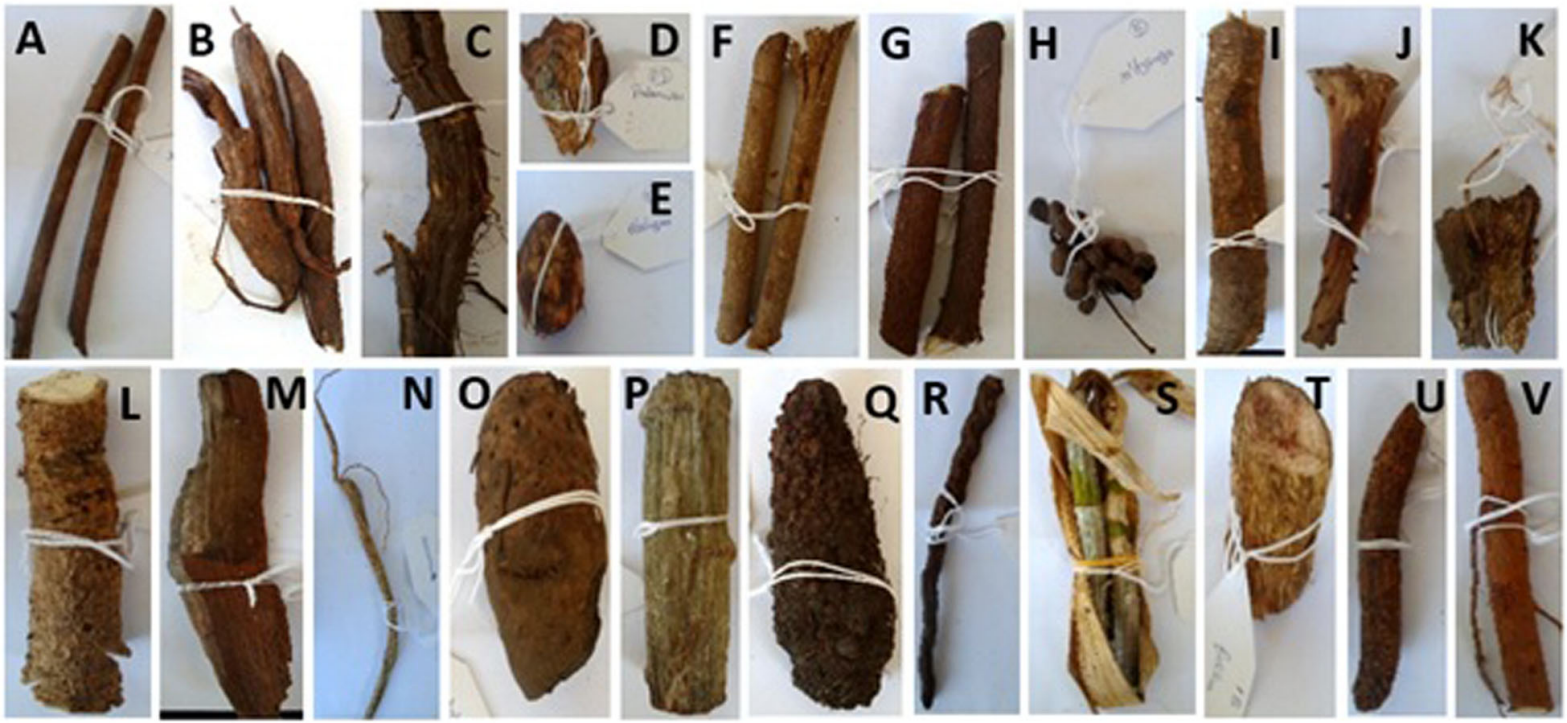
Photographs of some medicinal plant parts sold in Xipamanine, Xiquelene and Mazambane markets. **A** *Margaritaria discoidea* roots **B** *Elephantorrhiza elephantina* roots **C** *Tiliacora funifera* root **D** *Adenia gummifera* root **E** *Gladiolus* sp. bulb **F** *Terminalia sericea* root **G** *Gymnosporia heterophylla* root **H** *Dichrostachys cinerea* fruit **I** *Gymnanthemum coloratum* root **J** *Aloe marlothii* dry leaf piece **K** *Kedrostis* sp. root **L** *Strychnos spinosa* root **M** *Spirostachys africana* root **N** *Cucumis africanus* root **O** *Hypoxis hemerocallidea* corm **P** *Adenia gummifera* root **Q** *Hydnora abyssinica* rhizome **R** *Senna occidentalis* root **S** *Ansellia africana* stem **T** *Mucuna coriacea* root **U** *Ochna natalitia* root **V** *Garcinia livingstonei* root

**Fig. 2:**
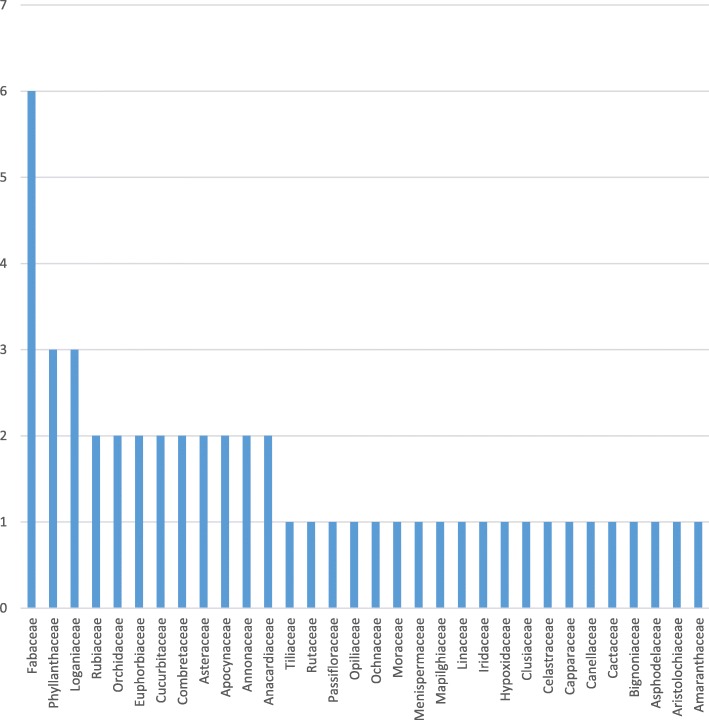
Frequency of botanical families and number of species

Figure 1 shows some of the medicinal plants sold in the markets. The most cited plants were *Terminalia sericea*, *Elephantorrhiza elephantina, Tiliacora funifera* and *Hypoxis hemerocallidea* with RFCs of 0.87, 0.80, 0.60 and 0.53, respectively. The plants belonged to 32 families, and the most frequent was Fabaceae with 6 species (Fig. 2). Some plants had more than one local name (e.g. *Senna occidentalis*, locally known as “Nhokane”, “Ndlha nhoka”, “Ndlha nhokane”, “Nhokane utsongo” and “Nhokane uculo”), that is related to the disease treated. “Nhokane”, meaning “roundworm”, is mainly used for the treatment of helminthiases. *Hydnora abissinica,* specie cited to treat UTI, helminthiases and internal wounds, was first recorded in southern Mozambique in a previous study [20]. It is well known by traders and traditional healers, therefore widely used within traditional medicine in southern Africa. This plant is rarely found by botanicals [20]. Phytochemical studies showed high tannin concentration in rhizomes which imparts a strong astringency and this may explain its efficacy in treating ailments of the digestive tract [21].

Fourteen plant materials could not be determined to family and species level in this study because these plant species were not available in the markets during the study period. It is more so important that in view of their ethnopharmacological importance these plant species deserve future attention for determination purpose.

Information on plant phytochemical studies and biological assays allows to know if their traditional use is validated. This information will be useful for defining subsequent studies of these plants.

**Fig. 3:**
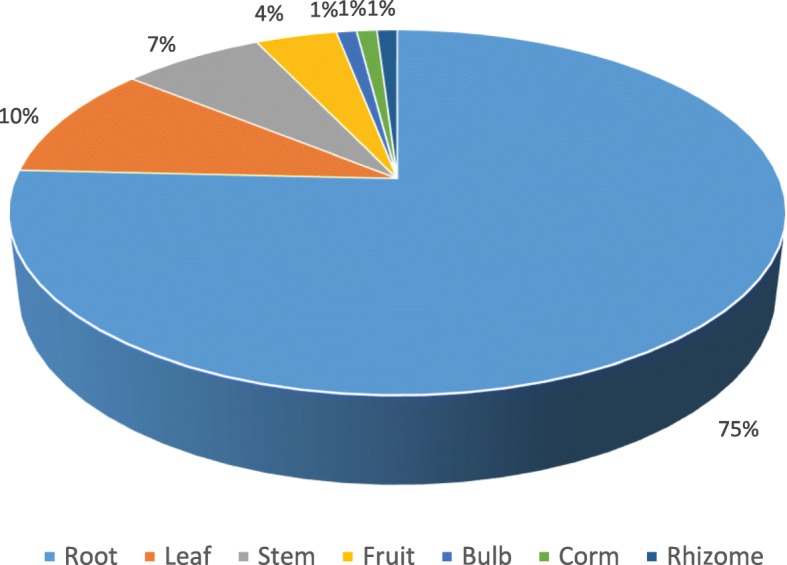
Frequency of sold plant parts

**Fig. 4:**
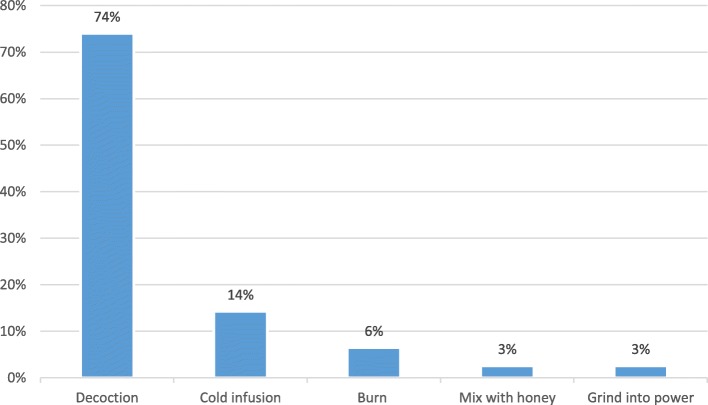
Frequency of medicinal plant preparation methods

Table 2 summarizes the ethnobotanical information collected in the markets regarding medicinal plant sold for treatment of bacterial and parasitic diseases. Roots were the most often sold and used parts (75%) (Fig. 3). The most cited diseases were helminthiases (28%) and diarrhea (18%) (Table 3). Decoction was the most common preparation method (74%) (Fig. 4), and oral administration route was the most common (79%), followed by topical (17%) and anal administration (4%). FL calculation for the most sold species and diseases is available on Table 4.

**Table 2:** Medicinal plants sold for treatment of bacterial and parasitic diseases in Xipamanine, Xiquelene and Mazambane markets, Mozambique

Scientific name / voucher no	Family	Local name	Parts used	Ailment treated	Preparation	Administration	RFC	UV
*Abrus precatorius* L. 4336	Fabaceae	Sissana	Root	Helminthiases	Decoction	Oral	0.07	0.07
*Acanthospermum hispidium* DC. 2624	Asteraceae	Chinamane	Leaf	Wounds	Cold infusion	Topical	0.07	0.07
*Acridocarpus natalitius* A.Juss. Z445	Mapilghiaceae	Mabope	Root	Diarrhea (bloody or not), haemorrhoids	Decoction	Oral	0.13	0.13
*Adenia gummifera* (Harv.) Harms MxiqDFB09	Passifloraceae	Pindevemmushay	Root	Internal wounds, helminthiases, tuberculosis^a^	Decoction	Oral	0.27	0.20
*Aloe marlothii* A. Berger JK7331	Asphodelaceae	*Aloe vera*, Mangane	Leaf	Wounds	Burn and apply the hot sap or powder	Topical (cover or wash)	0.40	0.27
				Diarrhea, UTI	Decoction	Oral		
				Cough	Mix with honey			
*Annona senegalensis* Pers. 1644	Annonaceae	Rompfha	Root	Wounds	Cold infusion	Topical -wash	0.13	0.13
				Cough	Decoction	Oral		
*Ansellia africana* Lindl. NM262	Orchidaceae	Phakama la hlanga / Phakama	Stem, leaves	Cough, tuberculosis	Decoction	Oral	0.20	0.13
*Ansellia* sp. K8132	Orchidaceae	Phakama lankulho	Stem	Cough	Decoction	Oral	0.07	0.07
*Artabotrys brachypetalus* Benth. 8198	Annonaceae	N’tita	Root	Helminthiases	Decoction	Oral	0.13	0.07
*Bridelia cathartica* Bertol. K8084	Phyllanthaceae	Thlathlangati	Root	Helminthiases	Decoction	Oral	0.13	0.13
			Leaf	UTI				
*Catunaregam spinosa* (Thunb.) Tirveng. KGD9651	Rubiaceae	Xirrole	Root	Helminthiases	Decoction	Oral	0.07	0.07
*Celosia* sp. 7390	Amaranthaceae	Vela valheka	Fruit	Furuncle	Burn into powder, add *Ricinus communis* seed oil	Topical	0.07	0.07
*Combretum molle* R.Br. ex G.Don. CM1520	Combretaceae	Xicucutse, Xiwondzwana	Root	Diarrhea, dysentery, helminthiases, UTI, wounds	Decoction	Oral	0.20	0.33
*Croton* sp. 3724	Euphorbiaceae	Tchequelanhama	Root	Epilepsy	Decoction	Oral	0.07	0.07
*Cucumis africanus* L.f. MxiqDFB11	Cucurbitaceae	Chiracarane	Root	Helminthiases, Schistosomiasis	Decoction or Cold infusion for 15 min	Oral	0.20	0.13
					Cold infusion	Rectal		
*Dichrostachys cinerea* (L.) Wight & Arn. 4166	Fabaceae	Tsenga	Fruit	Wounds	Burn into powder	Topical	0.07	0.07
*Elephantorrhiza elephantina* (Burch.) Skeels MxipDFB01	Fabaceae	Xivurai	Root	Helminthiases, diarrhea (bloody or not), cough, tuberculosis, dysentery, haemorrhoids	Decoction	Oral	0.80	0.40
*Garcinia livingstonei* T. Anderson MxiqDFB20	Clusiaceae	Bimbe, Mahimbe	Root and stem	Helminthiases, diarrhea, cough, dysentery	Decoction	Oral	0.47	0.27
*Gladiolus* sp. PSM1139	Iridaceae	Halahingwa	Bulb and root	Diarrhea, dysentery	Decoction	Oral	0.20	0.27
				Helminthiases, Schistosomiasis	Cold infusion	Rectal		
*Grewia sulcata* Mast. 8270	Tiliaceae	Chiuane	Root	Cough	Decoction	Oral	0.07	0.07
*Gymnanthemum coloratum* (Willd.) H.Rb. & B. Kahn MxipDFB05	Asteraceae	Nhathelo, Palhakufa	Root	Helminthiases	Decoction	Oral	0.07	0.07
*Gymnosporia heterophylla* (Eckl. & Zeyh.) Loes MxiqDFB03	Celastraceae	Xihlangua	Root	Diarrhea, dysentery	Decoction	Oral	0.27	0.13
*Heinsia crinita* (Afzel.) G. Taylor KGD9652	Rubiaceae	Xissindze	Root	Helminthiases	Decoction	Oral	0.07	0.07
*Hugonia orientalis* Engl. PC2349	Linaceae	Congulutamute	Root	Diarrhea	Decoction	Oral	0.13	0.13
				Wounds	Cold infusion	Topical - wash		
*Hydnora abyssinica* A.Br. MxipDFB14	Aristolochiaceae	Mavumbule	Rhizome	UTI, helminthiases, internal wounds	Decoction	Oral	0.20	0.20
*Hypoxis hemerocallidea* Fisch., C.A.Mey. & Avé-Lall. MxiqDFB4	Hypoxidaceae	Batata africana	Corm	Helminthiases, diarrhea (bloody or not), dysentery, wounds (internal or external), UTI, haemorrhoids	Decoction or Cold infusion	Oral	0.53	0.40
*Kedrostis* sp. MxiqDFB06	Cucurbitaceae	Dema amarelo, Dema	Root	Diarrhea, helminthiases, UTI, wounds	Decoction or Cold infusion	Oral	0.27	0.27
*Kigelia africana* (Lam.) Benth. 9781	Bignoniaceae	Mpfungura	Fruit	Deep wounds	Burn into powder, add *R. communis* seed. Cover the wound with honey first.	Topical	0.07	0.07
*Maclura africana* (Bureau) Corner GD617	Moraceae	Npumbulu	Root	Helminthiases	Decoction	Oral	0.07	0.07
*Maerua juncea* Pax PM954	Capparaceae	Chipinga	Root	Helminthiases	Decoction	Oral	0.13	0.07
*Mangifera indica* L. 7736	Anacardiaceae	Mangueira	Root	Helminthiases	Decoction	Oral	0.07	0.07
*Margaritaria discoidea* (Baill.) G.L.Webster MxiqDFB01	Phyllanthaceae	Xindikwe, Sinderane	Root	UTI, tuberculosis, cough	Decoction	Oral	0.20	0.20
*Mucuna coriacea* Baker MxiqDFB18	Fabaceae	Fethla	Root	Helminthiases, internal wounds	Decoction	Oral	0.13	0.13
*Ochna natalitia* (Meisn.) Walp. MxiqDFB19	Ochnaceae	Mathlanganisso	Root	Helminthiases, tuberculosis	Decoction	Oral	0.13	0.13
*Opilia amentacea* Roxb. GKD1159	Opiliaceae	Magunthlo	Root	Helminthiases	Decoction	Oral	0.07	0.07
*Opuntia ficus-indica* (L.) Mill. 10,485	Cactaceae	Xihaca	Stem	Cough	Cut and make syrup with honey	Oral	0.07	0.07
*Ozoroa obovata* (Oliv.) R. Fern. & A. Fern. 7771	Anacardiaceae	Chinungo	Root	Wounds	Cold infusion	Topical - wash	0.07	0.07
*Phyllanthus reticulatus* Poir. 8395	Phyllanthaceae	Tetenha	Root	Diarrhea	Decoction	Oral	0.07	0.07
*Secamone punctulata* Decne. 9588	Apocynaceae	Ximufane	Root	Diarrhea	Decoction	Oral	0.07	0.07
*Senna occidentalis* (L.) Link 3269	Fabaceae	Nhokane, Ndlha nhoka, Ndlha nhokane, Nhokane tsongo, Nhokane uculo	Root	Helminthiases, diarrhea	Decoction	Oral	0.40	0.13
*Senna petersiana* (Bolle) Lock 4301	Fabaceae	Nembe-nembe uculo	Root	Helminthiases, epilepsy	Decoction	Oral	0.13	0.13
*Spirostachys africana* Sond. MmazDFB03	Euphorbiaceae	Mubhandwa, Chilangamalho	Root and stem	Diarrhea, epilepsy, dysentery	Decoction	Oral	0.33	0.20
*Strychnos decussata* (Pappe) Gilg 3050	Loganiaceae	Xinkwakwani	Root	Helminthiases	Decoction	Oral	0.07	0.07
*Strychnos henningsii* Gilg 1740	Loganiaceae	Manono	Root	Abdominal pain	Cold infusion or decoction	Oral	0.13	0.13
			Root bark	Diarrhea with pain				
*Strychnos spinosa* Lam. 1738	Loganiaceae	Massala	Root	Helminthiases	Decoction	Oral	0.13	0.07
*Tabernaemontana elegans* Stapf 8923	Apocynaceae	Ncahlu	Root	Diarrhea, wounds, UTI	Decoction	Oral	0.20	0.20
*Terminalia sericea* Burch. ex DC. MxipDFB02	Combretaceae	Conola	Root, leaves	Diarrhea (bloody or not), dysentery, helminthiases, haemorrhoids	Decoction	Oral	0.87	0.33
			Root bark	Wounds	Dry and grind into power	Topical		
*Tiliacora funifera* (Miers) Oliv. MmazDFB01	Menispermaceae	Xiwizila	Root	Helminthiases, diarrhea	Decoction	Oral	0.60	0.13
*Warburgia salutaris* (G.Bertol.) Chiov. 10,615	Canellaceae	Xibaha	Root bark	Mouth ulcers, cough	Decoction	Oral	0.13	0.13
*Zanthoxylum capense* (Thunb.) Harv. K8014	Rutaceae	Manunguane	Root	UTI, helminthiases	Decoction	Oral	0.13	0.13
Unidentified	–	Xipenele	Root	Helminthiases	Decoction	Oral	0.07	0.07
Unidentified	–	Wutambuti	Root	UTI	Decoction	Oral	0.07	0.07
Unidentified	–	Tsatsalane	Leaf	Helminthiases	Decoction	Oral	0.07	0.07
Unidentified	–	Towane	Root	Diarrhea	Decoction	Oral	0.07	0.07
Unidentified	–	Tchongo	Root	Helminthiases	Decoction	Oral	0.07	0.07
Unidentified	–	Nicungo	Root	Wounds	Grind into powder	Topical	0.07	0.07
Unidentified	–	Nhanho	Root	Haemorrhoids	Decoction or burn into powder	Rectal	0.07	0.07
Unidentified	–	Nhacutslwani	Root	Diarrhea	Decoction	Oral	0.07	0.07
Unidentified	–	Nandzelate	Leaf	UTI	Decoction	Oral	0.07	0.07
Unidentified	–	Massoliza	Root	Helminthiases	Decoction	Oral	0.07	0.07
Unidentified	–	Magazine	Root	Diarrhea	Decoction	Oral	0.07	0.07
Unidentified	–	Magaranhaca	Root	Epilepsy	Decoction	Oral	0.07	0.07
Unidentified	–	Lilhatana	Root	Helminthiases	Decoction	Oral	0.07	0.07
Unidentified	–	Chepa	Root	Wounds	Cold infusion	Topical – wash	0.13	0.13
				Cough	Decoction	Oral		

^a^Tuberculosis of the respiratory system

**Table 3:** Most cited diseases by the informants by medical category and disease

Medical category^a^	Disease	n	%
Certain infectious or parasitic diseases	Helminthiases	33	28%
	Dysentery	8	7%
	Tuberculosis^b^	5	4%
	Schistosomiasis	2	2%
Diseases of the digestive system	Diarrhea	21	18%
	Haemorrhoids	5	4%
	Abdominal Pain	1	1%
Injury, poisoning or certain other consequences of external causes	Wounds	19	16%
Symptoms or signs involving the respiratory system	Cough	11	9%
Diseases of the genitourinary system	UTI	11	9%
Diseases of the nervous system	Epilepsy	4	3%

^a^The medical categories were found in the International Classification of Diseases 11th Edition from the WHO (https://icd.who.int/en)

^b^Tuberculosis of the respiratory system

**Table 4:** FL values for the most cited medicinal plants

Plant	Disease	Np	Ns	FL
*Terminalia sericea* Burch. ex DC.	Diarrhea	10	11	0.91
	Helminthiases	2	11	0.18
	Haemorrhoids	1	11	0.09
	Wounds	1	11	0.09
	Dysentery	1	11	0.09
*Elephantorrhiza elephantina* (Burch.) Skeels	Diarrhea	7	8	0.88
	Cough	3	8	0.38
	Helminthiases	1	8	0.25
	Tuberculosis	1	8	0.13
	Dysentery	1	8	0.13
	Haemorrhoids	1	8	0.13
*Tiliacora funifera* (Miers) Oliv.	Helminthiases	5	9	0.56
	Diarrhea	4	9	0.44
*Hypoxis hemerocallidea* Fisch. & C.A. Mey	Diarrhea	6	8	0.75
	Wounds	2	8	0.25
	Helminthiases	1	8	0.13
	Dysentery	1	8	0.13
	UTI	1	8	0.13
	Haemorrhoids	1	8	0.13

The vendors did not have concrete information about the time period in which plant-based preparations could be stored. Some informants said that the decoctions should be consumed until boiled for the third time. Other vendors said that the decoctions should be consumed until they stop having a bitter taste. Many reported that decoctions can be stored for 2 to 6 days, while some stated that they can be stored for 1 month if kept on the fridge. This is important because it contributes to the effectiveness of the remedies and can lead to microbial contamination if the remedy is poorly stored or kept for extended periods.

**Fig. 5:**
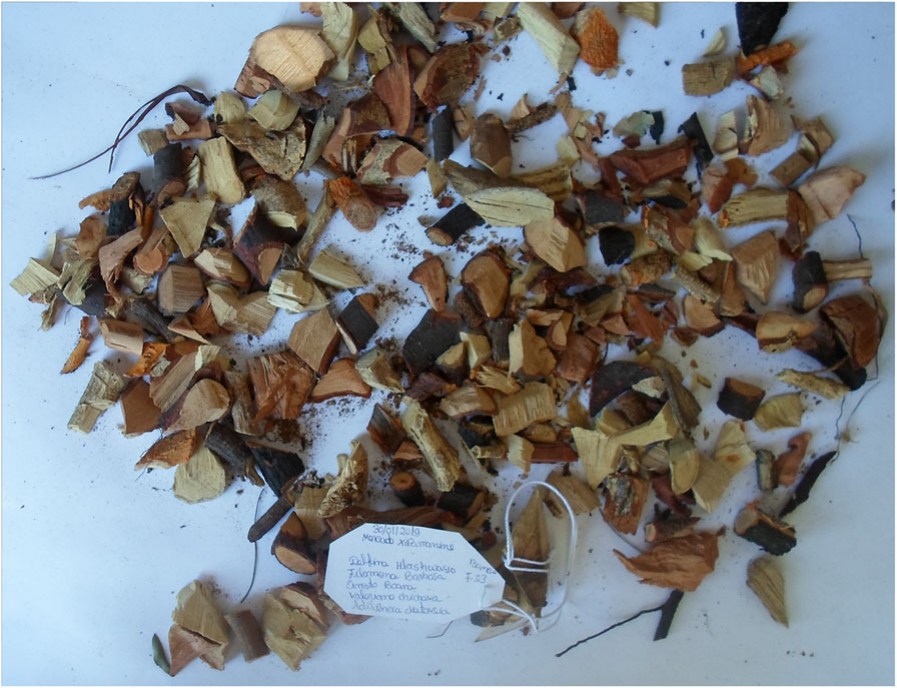
Plants sold in Xipamanine market

Most plants are prescribed and traded as mixtures previously cut and prepared by the vendors (Fig. 5). *Aloe marlothii, Celosia* sp.*, Cucumis africanus, Elephantorrhiza elephantina, Hypoxis hemerocallidea, Kedrostis* sp.*, Kigelia africana, Opuntia ficus-indica, Senna occidentalis, Strychnos henningsii, Terminalia sericea, Warburgia salutaris, “*Lilhatana*”, “*Nhanho*”, “*Nicungo*”* and *“*Tsatsalane*”* were the only plants that were traded separately by some informants, although other informants sell these plants in mixtures.

When we asked the vendors about the price of their products, many reported that it depends on the consumer’s buying capacity, i.e. if the consumer does not have financial resources, they will be charged a reduced price. Vendors affirmed that they are working to heal people and for this reason money does not matter much. Medicinal plant prices were very low and range from 10 MZN (0.16 USD) to 150 MZN (2.43 USD). The average prices range from 10 MZN to 50 MZN (0.81 USD). The two highest priced plants are *Warburgia salutaris*, whose 15 cm piece of stem bark costed 100 MZN (1.62 USD) and *Mangifera indica* root, which was 150 MZN (2.43 USD). An herbal mix used to treat cough had the highest price of 250 MZN (4.05 USD). The mixture contained cut roots and leaves of the following plants: *Annona senegalensis*, *Margaritaria discoidea*, *Grewia sulcata*, *Ansellia africana*, *Elephantorrhiza elephantina* and “Chepa*”* (unidentified specie).

### Practices associated with the consumption of the remedies

Some unusual practices are associated with remedies consumption, for example, *W. salutaris* stem bark decoction has to be dunk at hot-to-warm temperature on a cold day and at room temperature in a hot day for cough treatment. For helminthiases treatment, *Cucumis africanus* root decoction has to be drunk only when the sky is clear. These and other practices are common in traditional African medicine and sometimes refer to spiritual, empirical and traditional matters.

It was also noted that traditional medicine in Mozambique is not dissociated nor against conventional medicine, since many vendors advise the consumers to seek for medical services if the disease does not heal through medicinal plants.

Some informants cited other remedies that occur in the city and nearby places but are not sold. Burned snail shell (*Achatina fulica*) and *Ricinus communis* seed oil are mixed for treatment of furuncles. Burned *Solanum tuberosum* (known as “batata reno*”*) tuber is also added to *R. communis* seed oil for the same purpose. *Sclerocarya birrea* (“canhueiro”) and *Anacardium occidentale* (“cajueiro*”*) root barks are decocted to treat cough. Cold infusion of *Allium sativum* (known as “xinhalane” or “alho”) bulb is taken to treat helminthiases.

Some plants are also used for patient recovery after medicinal treatment. For example, *Elephantorrhiza elephantina* root and *Ximenia americana* (locally known as “tunduluka*”*) root bark are used to treat weakness that results from helminthiases infection. A cold infusion is made and the filtrate is used to make corn porridge that is ingested for one week.

## Discussion

A large diversity of plants was recorded as sold for treatment of bacterial and parasitic diseases. It was also found that there is still a high trade of plants in Maputo markets, and a high demand by the population. Most of the plants cited in this study were documented in one previous study done in 2004, although some vernacular names differ [6].

The vendors did not provide detailed information about how they collect or obtain the plants, i.e. if they collect themselves or acquired by other means. However, many explained that the plants were collected in many districts from Maputo city and province, namely: KaTembe, Boane, Manhiça, Moamba, Marracuene and Matutuine, and in Gaza and Inhambane, the nearby provinces also located in southern Mozambique. According to one of the vendors, *W. salutaris* was the only species collected cross border in South Africa. Other vendors affirmed that it was collected from Moamba district in Maputo province and at Inhambane province. This information is worrisome because this plant is endangered according to the IUCN Red List of Threatened Species 1998 [22].

It was important to record where the plants are harvested, mainly in Maputo province. The majority of districts in the province are rapidly getting urbanized because of house constructions. Therefore, there is an increased risk of deforestation and this can threaten the availability of the medicinal plants. Moreover, it might be difficult for vendors to obtain the plants in the future.

All of the most cited plants in this study had a high FL for diarrhea. The higher value of FL was found for *Terminalia sericea* (=0.91), followed by *Elephantorrhiza elephantina*, *Hypoxis hemerocallidea* and *Tiliacora funifera* with values of 0.88, 0.75 and 0.44, respectively. All these plants are native from Africa and Mozambique.

*T. sericea* is widely used in the traditional medicine in this continent [23]. It was among the most traded medicinal plants in Mpumalanga Province, South Africa [24]. Among other medicinal uses reported in South Africa, roots were commonly used to treat diarrhea and infectious diseases [25]. Phytochemical studies were conducted and most of the active ingredients were isolated from the roots and stem bark [23].

Several in vitro studies were conducted for *T. sericea* [26–28]. For example works on intestinal infection-causing bacteria showed that *T. sericea* is effective against various bacteria such as *Micrococcus luteus*, *Enterobacter aerogenes*, *Streptoccocus pyogenes* and *Staphylococcus aureus* [26, 27]. Other study has reported the activity of ethyl acetate root extract against *Bacillus subtilis* and *Escherichia coli*, with MICs of 0.3 and 1.5 mg/ml, respectively. These data shows that *T. sericea* is effective against various pathogenic microorganisms, including enteric pathogens [28].

*Elephantorrhiza elephantina*, was also cited in other South African studies for the treatment of diarrhea, helminthiases but results from this study revealed other diseases including cough, tuberculosis, dysentery and haemorrhoids. Active principles have already been isolated and biological activity against several pathogens has been studied, with positive results [29, 30].

*Hypoxis hemerocallidea*, also known as a miracle plant because it has various therapeutic uses has been used in traditional African medicine for many years. The plant has been extensively studied at the laboratory level for a variety of purposes beyond those reported in this research [31]. The plant had higher FL for diarrhea. It has also been cited for the treatment of other diseases such as wounds, helminthiases dysentery, Urinary Tract Infection (UTI) and haemorrhoids. The antibacterial activity of corm and leaves extracts was positive against enteric bacteria and other organisms [32].

*Tiliacora funifera*, cited for treatment of helminthiases and diarrhea, has anti-plasmodium activity due to alkaloids [33]. Ethnobotanical studies report that *T. funifera* root contributes to women’s fertility and the leaves are used for treatment of facial skin problems [34]. The sap of the leaves is used in herbal remedies to prevent insanity in Congo, while in Ghana it is used to treat gastric fever, hernia and menstrual disorders [35]. This plant was also cited by many vendors in the previous study done in the study sites [6].

No recent laboratory studies were found for *T. funifera* and ethnobotanical studies do not often report the antidiarrheal and antihelmintic use of the plant. Thus, in vitro studies to validate medicinal use of this species are crucial. Nevertheless, studies reporting antimicrobial activity of other *Tiliacora* species have been found [36].

Curiously, one vendor said that *T. funifera* cures any disease and is more effective than African potato (*Hypoxis hemerocallidea*). This is important to note once *H. hemerocallidea* is consistently harvested and widely used to treat AIDS-related illnesses in the African continent. Attention should be paid to overexploitation of these plants in their natural habitat.

*Hydnora abissinica,* specie cited to treat UTI, helminthiases and internal wounds, was first recorded in southern Mozambique in a previous study [20]. It is well known by traders and traditional healers, therefore widely used within traditional medicine in southern Africa. This plant is rarely found by botanicals [20]. Phytochemical studies showed high tannin concentration in rhizomes which imparts a strong astringency and this may explain its efficacy in treating ailments of the digestive tract [21].

Fourteen plant materials could not be determined to family and species level in this study because these plant species were not available in the markets during the study period. It is more so important that in view of their ethnopharmacological importance these plant species deserve future attention for determination purpose.

Information on plant phytochemical studies and biological assays allows to know if their traditional use is validated. This information will be useful for defining subsequent studies of these plants.

## Conclusions

Many plants are sold for the treatment of bacterial and parasitic diseases in the three main medicinal plants markets in Maputo city. Most of the plants are found in Maputo province forests. Access to plants in Maputo is becoming difficult due to increasing urbanization. Some endangered plants are still being sold, which is something to be aware and to develop mitigation strategies.

Fourteen plant materials could not be determined to family and species level. Neverthless these plant species deserve future attention for determination purpose.

Medicinal plants are still commonly used and traded, even in large urban centers such as Maputo city. This data shows that the population still uses traditional medicine to treat their diseases.

This register of medicinal plants is very important once an erosion of knowledge is taking place. Some plants have already been studied in vitro for biological activity but other plants should be studied in order to evaluate their efficacy. In future new drug candidate molecules can be developed based on these plants against multidrug resistant strains.

## Data Availability

All datasets used and/or analyzed during the current study are available from the corresponding author on reasonable request.

## Abbreviations

FL: Fidelity Level
RFC: Relative Frequency of Citation
UTI: Urinary Tract Infection
UV: Use Value
